# A machine learning-based model for a dose point kernel calculation

**DOI:** 10.1186/s40658-023-00560-9

**Published:** 2023-06-26

**Authors:** Ignacio Scarinci, Mauro Valente, Pedro Pérez

**Affiliations:** 1 Instituto de Física Enrique Gaviola (IFEG), CONICET, Av. Medina Allende s/n, 5000 Córdoba, Argentina; 2 Laboratorio de Investigación e Instrumentación en Física Aplicada a la Medicina e Imágenes de Rayos X (LIIFAMIRx), Facultad de Matemática, Astronomía, Física y Computación, Universidad Nacional de Córdoba, Av. Medina Allende s/n, 5000 Córdoba, Argentina; 3 Centro de Excelencia en Física e Ingeniería en Salud (CFIS) & Departamento de Ciencias Físicas, Universidad de la Frontera, Avenida Francisco Salazar 01145, 4811230 Temuco, Cautín Chile

**Keywords:** Beta emitters, Dose point kernel, Internal dosimetry, Machine learning

## Abstract

**Purpose:**

Absorbed dose calculation by kernel convolution requires the prior determination of dose point kernels (DPK). This study reports on the design, implementation, and test of a multi-target regressor approach to generate the DPKs for monoenergetic sources and a model to obtain DPKs for beta emitters.

**Methods:**

DPK for monoenergetic electron sources were calculated using the FLUKA Monte Carlo (MC) code for many materials of clinical interest and initial energies ranging from 10 to 3000 keV. Regressor Chains (RC) with three different coefficients regularization/shrinkage models were used as base regressors. Electron monoenergetic scaled DPKs (sDPKs) were used to assess the corresponding sDPKs for beta emitters typically used in nuclear medicine, which were compared against reference published data. Finally, the beta emitters sDPK were applied to a patient-specific case calculating the Voxel Dose Kernel (VDK) for a hepatic radioembolization treatment with $^{90}$Y.

**Results:**

The three trained machine learning models demonstrated a promising capacity to predict the sDPK for both monoenergetic emissions and beta emitters of clinical interest attaining differences lower than $10\%$ in the mean average percentage error (MAPE) as compared with previous studies. Furthermore, differences lower than $7 \%$ were obtained for the absorbed dose in patient-specific dosimetry comparing against full stochastic MC calculations.

**Conclusion:**

An ML model was developed to assess dosimetry calculations in nuclear medicine. The implemented approach has shown the capacity to accurately predict the sDPK for monoenergetic beta sources in a wide range of energy in different materials. The ML model to calculate the sDPK for beta-emitting radionuclides allowed to obtain VDK useful to achieve reliable patient-specific absorbed dose distributions required short computation times.

**Supplementary Information:**

The online version contains supplementary material available at 10.1186/s40658-023-00560-9.

## Introduction

Personalized medicine advances have significantly enhanced the efficacy of therapeutic and palliative treatments for several diseases [1–3]. The introduction of theranostic therapies, combining therapeutic and diagnostic imaging using a single radiopharmaceutical, has increased interest in radiopharmaceuticals in various cancer treatments, particularly in nuclear medicine [4–6]. The capacity to generate molecular imaging, such as SPECT or PET, used for treatment procedures for real-time monitoring of the whole radiopharmaceutical metabolization process, together with the capacity to obtain anatomical images alongside molecular imaging (SPECT/CT, PET/CT, PET/MRI), allows for significant improvements in dosimetric estimates both before and after treatment [7–9]. Thus, theranostic enables patient-specific dosimetric calculations based on molecular and anatomical imaging, improving radionuclide treatment effectiveness and safety [10, 11].

Several approaches for internal dose estimation in nuclear medicine procedures have been developed, such as Monte Carlo (MC) transport simulation [12, 13], S-value estimation [14, 15], and dose point kernel (DPK) convolution [16, 17]. The MC method is the most precise dosimetric calculation approach, but it requires many computational resources and long computation times, making it sometimes inappropriate for clinical usage. On the other hand, methods such as S-values or DPK convolution allow for shorter computational times at the expense of lower computational accuracy. Thus, a tradeoff between computational time and the precision or accuracy of the dosimetric calculation is desirable.

Calculating the absorbed dose by convolution of DPKs requires a prior calculation of the DPKs. An extensive list of beta DPKs has been published [18–23]. Also, different methods for scaling DPKs to different media have been proposed since they are commonly calculated for water; then, DPKs for other media are obtained by applying different scaling factors [24, 25]. From the calculated beta DPKs, the so-called Voxel S-values (VSV) or Voxel Dose Kernel (VDK) can be obtained, which facilitate the absorbed dose calculation by convolution with the accumulated activity since they are represented in the form of three-dimensional matrices, and the convolution product can be solved in a discrete form, simplifying the calculation of the absorbed dose [26].

Artificial intelligence (AI) disrupted many fields of medical sciences due to computational advances that occurred over the last decade in terms of innovative hardware and software [27, 28], also impacting nuclear medicine [29–31]. In the internal dosimetry field, some Deep Learning (DL)-based models have been developed to predict patient-specific doses based on anatomic and metabolic imaging information; for example, Göetz et al. [32] propose a U-Net Deep Neural Network (DNN) that takes as input the activity distribution and the density map, the input is an array of $80 \times 80 \times 11$, and the output is the map of dose corresponding to the dosimetry of the middle slice in the inputs arrays; Akhavanallaf et al. [33] propose a DNN to predict the distribution of deposited energy due to 18FDG representing specific 3*D* voxelized S-values and calculate the absorbed dose by convolution of this with the activity distribution; Li et al. [34] train a model of convolutional neural network that learns only the difference between the true dose rate map (calculated by MC) and DVK dose rate map with density scaling, the input to the DNN is an array of size $512 \times 512 \times 11$, and the out is an array of $512 \times 512$ that corresponded to the dosimetry of the middle slice in the input arrays.

The calculation of DPKs can be considered a multi-target regression problem in which from the characteristics of the medium, such as chemical composition and density, as well as physical characteristics, such as the initial energy of the source electrons and the range of these in the medium considered, the DPK value as a function of the distance *r* to the source is predicted as a target variable [35]. These types of models are widely used in problems of ecological modeling [36], healthcare [37], environmental [38], and drug discovery [39].

The naive solution is the Multi-Target Regressor Stacking (MTRS) [40], a stack of regressor for each target variables. However, this does not consider that the target variables are related and depend on each other. Other models that capture the relationship between the target variables are, for example, Regressor Chains (RC) [40], in which a series of base regressors are chained to predict the variable of interest. Each model in the chain uses the predictions of the previous model as input, and this model allows transforming linear regression algorithms from single-target to multi-target. Multi-Target Support Vector Machine Regressors [41, 42] is a multi-target version of widely used support vector machine regressor [43], and this model is powerful, but the main disadvantage of this model is the complexity time which increases with the number of samples. Random forest regressors show a great capacity to solve this kind of problem [44]. However, the interpretability of this model is more complex than linear regressors. Finally, artificial neural networks (ANN) [45] have the advantage of representing non-linear problems, but the search for optimal hyperparameters is an arduous task.

This study presents an RC model with linear regressors as the base regressors for predicting monoenergetic sDPK. In addition, three regularization methods such as Ridge [46], Lasso [47], and ElasticNet [48] are implemented. The Ridge regression uses an L2-norm regularization to constrain model coefficients. The Lasso regression uses an L1-norm, which produces some coefficients that are exactly zero, so this works as a coefficient shrinkage and feature selection. The Elastic Net regression model combines L1-norm with L2-norm penalty functions, simultaneously performing features selection and coefficient regularization. Moreover, this allows the linear regression model to tolerate the multicollinearity of predictors [49]. We focus on these base regressors because they are of lower computational complexity [50] and using a regressor chains with linear regressors is an interpretable approach.

From the monoenergetic sDPKs calculated by the regressor chains, a methodology for estimating the sDPK and the VDK for beta-emitting radionuclides is determined. A dosimetric application is performed for $^{90}$Y radionuclide as a test case; then, the absorbed map dose is determined by the convolution of the cumulative activity per voxel with the VDK calculated from the sDPK and MC volume integration. The two approaches are compared and validated by gamma index testing.

This study reports on the implementation of three ML models to predict the sDPK for beta emitter nuclear medicine nuclides as well as to achieve patient-specific dosimetry quantitatively comparable to Monte Carlo calculations, but requiring much less computation time being able to provide reliable 3D dose distributions in a few minutes.

## Materials and methods

### Radionuclides and materials

Table 1 summarizes some beta-emitting radionuclides usually applied in treating different diseases [51–55]. The overall maximum energy emitted is 2275.6 keV for $^{90}$Y; therefore, energy values between 10 and 3000 keV were considered for monoenergetic beta sDPK calculation.

A method for calculating the sDPK for a spectral emission of beta particles from the sDPK for a monoenergetic beta emission is depicted in the following section.

**Table 1:** Beta-emitting radionuclides applied in treating diseases

Radionuclide	Half-life [days]	$E_{\rm avg}$($E_{\rm max}$)[keV]	Therapeutic indication
$^{89}$Sr	50.6	587.1 (1502.2)	Relief of pain skeletal metastases
$^{90}$Y	2.7	932.4 (2275.6)	Hepatocellular cancer and liver metastasis Non-Hodgkin’s lymphoma
$^{131}$I	8.0	191.6 (806.9)	Hyperthyroidism, differentiated thyroid cancer Non-Hodgkin’s lymphoma
$^{177}$Lu	6.7	148.8 (496.8)	Neuroendocrine tumors Prostate tumors
$^{186}$Re	3.7	359.2 (1072.7)	Relief of pain, skeletal metastases
$^{188}$Re	16.7	795.4 (2120.4)	Relief of pain, skeletal metastases

As usual, the studied materials were divided into two sets: the training and the testing sets. Material information in terms of Hounsfield Unit (HU), as described by Schneider et al. [56], was used to define the training set composition, and mass densities were assessed using the mean value of the HU considered range [57]. Subsequently, the following materials were used as testing compositions: air, lung, soft tissue, and cortical bone, according to the ICRP Publication 89 [58]. Tables 2 and 3 summarize the composition of the training and testing set.

**Table 2:** Material composition in weight fraction and density $[g/\text {cm}^{3}]$ of training dataset

Material	H	C	N	O	Na	Mg	P	S	Cl	Ar	K	Ca	Density
HU-950			0.755	0.232						0.013			0.0279
HU-120	0.103	0.105	0.031	0.749	0.002		0.002	0.003	0.003		0.002		0.4810
HU-83	0.116	0.681	0.002	0.198	0.001			0.001	0.001				0.9572
HU-53	0.113	0.567	0.009	0.308	0.001			0.001	0.001				0.9581
HU7	0.108	0.356	0.022	0.509	0.000		0.001	0.002	0.002				1.0108
HU18	0.106	0.284	0.026	0.578	0.000		0.001	0.002	0.002		0.001		1.0030
HU80	0.103	0.134	0.030	0.723	0.002		0.002	0.002	0.002		0.002		1.0591
HU120	0.094	0.207	0.062	0.622	0.006		0.000	0.006	0.003		0.000		1.1187
HU200	0.095	0.455	0.025	0.355	0.001		0.021	0.001	0.001		0.001	0.045	1.1111
HU300	0.089	0.423	0.027	0.363	0.001		0.030	0.001	0.001		0.001	0.064	1.1644
HU400	0.082	0.391	0.029	0.372	0.001		0.039	0.001	0.001		0.001	0.083	1.2236
HU500	0.076	0.361	0.030	0.380	0.001	0.001	0.047	0.002	0.001			0.101	1.2828
HU600	0.071	0.335	0.032	0.387	0.001	0.001	0.054	0.002				0.117	1.3420
HU700	0.066	0.310	0.033	0.394	0.001	0.001	0.061	0.002				0.132	1.4012
HU800	0.061	0.287	0.035	0.400	0.001	0.001	0.067	0.002				0.146	1.4604
HU900	0.056	0.265	0.036	0.405	0.001	0.002	0.073	0.003				0.159	1.5196
HU1000	0.052	0.246	0.037	0.411	0.001	0.002	0.078	0.003				0.170	1.5788
HU1100	0.049	0.227	0.038	0.416	0.001	0.002	0.083	0.003				0.181	1.6380
HU1200	0.045	0.210	0.039	0.420	0.001	0.002	0.088	0.003				0.192	1.6972
HU1300	0.042	0.194	0.040	0.425	0.001	0.002	0.092	0.003				0.201	1.7564
HU1400	0.039	0.179	0.041	0.429	0.001	0.002	0.096	0.003				0.210	1.8156
HU1500	0.036	0.165	0.042	0.432	0.001	0.002	0.100	0.003				0.219	1.8748
HU1600	0.034	0.155	0.042	0.435	0.001	0.002	0.103	0.003				0.225	1.9340

**Table 3:** Material composition in weight fraction and density $[g/\text {cm}^{3}]$ of testing dataset

Material	H	C	N	O	Na	Mg	P	S	Cl	Ar	K	Ca	Density
Air		0.0001	0.755	0.231						0.012			0.0012
Lung	0.101	0.102	0.028	0.757	0.001	0.0007	0.0008	0.002	0.002		0.001	0.00009	1.05
Soft Tissue	0.104	0.232	0.024	0.630	0.001	0.0001	0.001	0.002	0.001		0.002	0.0002	1.00
Cortical Bone	0.047	0.144	0.042	0.446		0.002	0.105	0.003				0.209	1.85

### Beta-emitting radionuclide sDPK

The electron-beta DPK is a function that represents the radial distribution of a specific absorbed fraction of dose in an infinite homogeneous medium due to a monoenergetic point source of beta or electron particles. A more useful form of DPK is the scaled sDPK, defined as

1 $$\begin{aligned} F\left( \frac{r}{r_0}\right) =4 \pi \rho r^2 r_0\Phi \left( r\right) \end{aligned}$$

where $\rho$ is the medium’s density, $r_0$ the range in the Continuous Slowing Down Approximation ($R_{\rm CSDA}$) approximation, and $\Phi (r)$ is the fraction of absorbed energy at distance *r*. The beta-emitting radionuclide sDPK can be defined as

2 $$\begin{aligned} F_{\beta }\left( \frac{r}{r_N}\right) =4 \pi \rho r^2r_N \Phi _{\beta }\left( r\right) \end{aligned}$$

where $r_N$ is the range for the maximum emission energy of the radioisotope and $\Phi _\beta (r)$ is the fraction of absorbed energy at a distance *r* [24]. Considering an infinite sphere of a homogeneous material with a source of beta particles in the center, the fraction of absorbed energy at a distance from the center is defined as [59]

3 $$\begin{aligned} \Phi _{\beta }\left( r\right) =\frac{\int _0^{E_{\max }}E_0\frac{{\rm d}I}{{\rm d}E_0}\Phi \left( r,E_0\right) {\rm d}E_0}{\int _0^{E_{\max }}E\frac{{\rm d}I}{{\rm d}E_0}{\rm d}E_0} \end{aligned}$$

and $\Phi _{\beta }(r,E)$ is the fraction of absorbed energy at a distance *r* to energy *E*. Equation 3 is approximated by

4 $$\begin{aligned} \Phi _{\beta }\left( r\right) =\frac{\sum _{\ j}^{\ }I_jE_{0j}\Phi _j\left( r,E_{0j}\right) }{E_{\rm eff}} \end{aligned}$$

where $I_j$ is the strength of the j-th group with mean energy $E_{0j}$, $\Phi (r,E_{0j})$ is the fraction of absorbed energy at a distance due to the j-th group of the spectrum and the effective energy is $E_eff=\sum _j I_j E_{0j}$. From Eqs. 2 and 4,

5 $$\begin{aligned} F\left( \frac{r}{r_N}\right) =\frac{\sum _j^4\pi \ \rho \ r^2r_N\ I_j\ E_{0j}\ \Phi _j\ \left( r,E_{0j}\right) }{E_{\rm eff}} \end{aligned}$$

Introducing Eq. 1 in 5, the sDPK of a betta-emitting radionuclide can be estimated from the sDPK for monoenergetic electron source as

6 $$\begin{aligned} F\left( \frac{r}{r_N}\right) =\frac{r_{N\sum _{\ j}^{\ }I_j}E_{0j}\frac{F_j\left( \frac{r}{r_{0j}},E_j\right) }{r_{0j}}}{E_{\rm eff}} \end{aligned}$$

Equation 6 states that knowing the sDPK for the monoenergetic source and the emission spectra of the radionuclide are required to obtain the sDPK for the radionuclide.

### DPK estimation by Monte Carlo simulations

The DPK for each material was obtained through MC simulations using the general-purpose software FLUKA version 2021.2.0, capable of calculating detailed radiation transport and energy deposition [60, 61]. FLUKA can simulate the whole track of several particles like photons, electrons, neutrons, and hadrons on a wide range of energies. It has been widely used for high-energy physics, experiencing an increasing application for medical physics purposes [62, 63]. FLUKA implements an original algorithm for treating multiple scattering on charge particles transport based on the Bethe improved Moliere’s theory [64].

FLUKA incorporates standard configurations which activate/deactivate by default various features according to the required physical model; in this study, the PRECISIOn default was applied, activating the electromagnetic interactions, the Rayleigh scattering, and the inelastic form factor corrections to Compton scattering and Compton profiles. The transport and production threshold was set on 1 keV for electrons and photons with initial energy under 100 keV, and it was set on 10 keV energies above 100 keV. Furthermore, single scattering was set up at boundaries for electron energies from 10 to 100 keV. Preliminary tests showed that 10 independent cycles of $10^6$ primary particles each cycle were the appropriate configuration to be set to obtain accurate results.

The phantom used for DPK calculations consists of 60 concentric spherical shells of homogeneous material whose outer radius is $1.5R_{\rm csda}$. Each shell has a thickness of $R_{\rm CSDA}/40$. The $R_{\rm CSDA}$ value is obtained using the fitting proposed by Tabata et al. [65], where the $R_{\rm CSDA}$ is calculated taking into account the effective atomic number $Z_{\rm eff}$ and the effective atomic weight $A_{\rm eff}$ of compound. Also, a monoenergetic electron source was positioned at the center of the spheres.

FLUKA provides the deposited energy $\delta E$ on each $\delta r$ thick shell. It is convenient to define the sDPK according to the results obtained from simulations and the initial kinetic energy $E_0$ expressed in MeV, and the range $R_{\rm CSDA}$ expressed in cm as [21]

7 $$\begin{aligned} F\left( \frac{r}{R_{\rm CSDA}}\right) =\frac{\delta E\left( r\right) /E_0}{\delta r/R_{\rm CSDA}} \end{aligned}$$

### sDPK estimation by ML

The problem of predicting monoenergetic sDPK from physical and chemical properties can be represented by a multivariate or multi-target regression [35]. Let *D* be a training dataset made up of *N* instances such that $D={(X_1,Y_1),\ldots ,(X_N,Y_N)}$. Likewise, each sample consists of an input array $X_N$ of dimensions *m* such that $X_i=(x_1,\ldots ,x_m)$ and a target array *Y* of sizes *k* such that $Y_i=(y_1,\ldots ,y_k)$. The problem is reduced to training a multi-target regressor model, which consists of finding a function *h* that assigns an array *Y* to each array *X*, that is

8 $$\begin{aligned} Y=h(X): h:{\mathbb {R}}^m \rightarrow {\mathbb {R}}^k \end{aligned}$$

The algorithm used was an RC [40]; this chain *M* was composed of *k* base regressors *m*, so that $M(m)=[M_1(m),\ldots ,M_i(m),\ldots ,M_k(m)]$. The dimension *k* is the same as the dimension of the target array *Y*. Firstly, a model $M_1$ is trained with all input features *X* and the first element of the target array $y_1$, then, a second model $M_2$ is trained with elements of *X* along with the first element $y_1$ as input features, and the second element of *Y* array $y_2$ is the target. Repeat this procedure until all *M* models for each element of array *Y* have been trained.

Three base regressors were studied: Ridge [46], Lasso [47], and Elastic Net [48] which use different regularized terms. The Ridge algorithm minimizes the residual sum of squares subject to bound on the L2-norm of the coefficients:

9 $$\begin{aligned} \min _w \Vert Xw-y\Vert _2^2+\alpha \Vert w\Vert _2^2 \end{aligned}$$

where $\alpha \geqslant 0$ is a constant, and $\Vert w\Vert _2^2$ is the L2-norm of the coefficient vector. The Lasso algorithm is a penalized least-squares method imposing an L1-penalty on the regression coefficients:

10 $$\begin{aligned} \min _w \Vert Xw-y\Vert _2^2+\alpha \Vert w\Vert _1 \end{aligned}$$

where $\alpha$ is a constant, and $\Vert w\Vert _1$ is the L2-norm of the coefficient vector. The Elastic Net algorithm penalized the least-squares method using a combination of both kinds of regularization:

11 $$\begin{aligned} \min _w \frac{1}{2n_{\rm samples}} \Vert Xw-y\Vert _2^2 + \gamma \alpha \Vert w\Vert _1 + \frac{\alpha (1-\gamma )}{2} \Vert w\Vert _2^2 \end{aligned}$$

where $\alpha$ and $\gamma$ are constants and $\Vert w\Vert _1$ and $\Vert w\Vert _2^2$ are the L1-norm and L2-norm of the coefficients vector, respectively.

The system was characterized by the following features of the source: energy $E_0[keV]$, the range $R_{\rm CSDA} \, [g/\text {cm}^2]$, density of medium material $\rho [g/\text {cm}^3]$, and the composition of the material in weight fraction for the elements considered (H, C, Na, Mg, P, S, Cl, Ar, K, Ca), and the monoenergetic sDPK was the target.

Two metrics used to evaluate the models were the coefficient of determination ($R^2$) and the Root Mean Square Error (RMSE), defined as

12 $$\begin{aligned} R^2(y,y')&=1-\frac{\sum _{i=1}^{n} (y_i-y'_i)^2}{\sum _{i=1}^{n} (y_i-{\bar{y}}_i)^2}\nonumber \\ \text {RMSE}(y,y')&=\sqrt{\frac{1}{n_{\rm samples}} \sum _{i=0}^{n_{\rm samples}-1}(y_i-y'_i)^2} \end{aligned}$$

where $y_i$ and $y'_i$ are i-th components of the sDPK calculated by MC and ML models, respectively.

The model predicts the monoenergetic sDPK for a given energy and chemical composition of the medium and applies Eq. 6 to obtain the sDPK for a given beta emitter in a particular medium.

### Benchmark evaluation of the calculated sDPK for beta-emitting radionuclides

Beta-emitting radionuclide sDPK calculated by Eq. 6 was benchmarked against previously reported results by Botta et al. [66] and Shiiba et al. [67]. Botta used FLUKA for simulating the sDPK, and Shiiba used PHITS (56). The radionuclides considered were $^{89}$Sr, $^{90}$Y, $^{131}$I, $^{177}$Lu, $^{186}$Re, and $^{188}$Re; the materials considered were water and compact bone. The Mean Absolute Percentage Error (MAPE) was used as a metric, defined as

13 $$\begin{aligned} \text {MAPE}(F,F')=\frac{100}{n_{\rm samples}} \sum _{i=1}^{n_{\rm shells}} \frac{\Vert F_i-F'_i\Vert }{\Vert F_i\Vert } \end{aligned}$$

where *F* was the reference sDPK and $F'$ the sDPK estimated by the ML model for the i-th shell and *n* is the number of samples.

### Dosimetry calculation

**Fig. 1:**
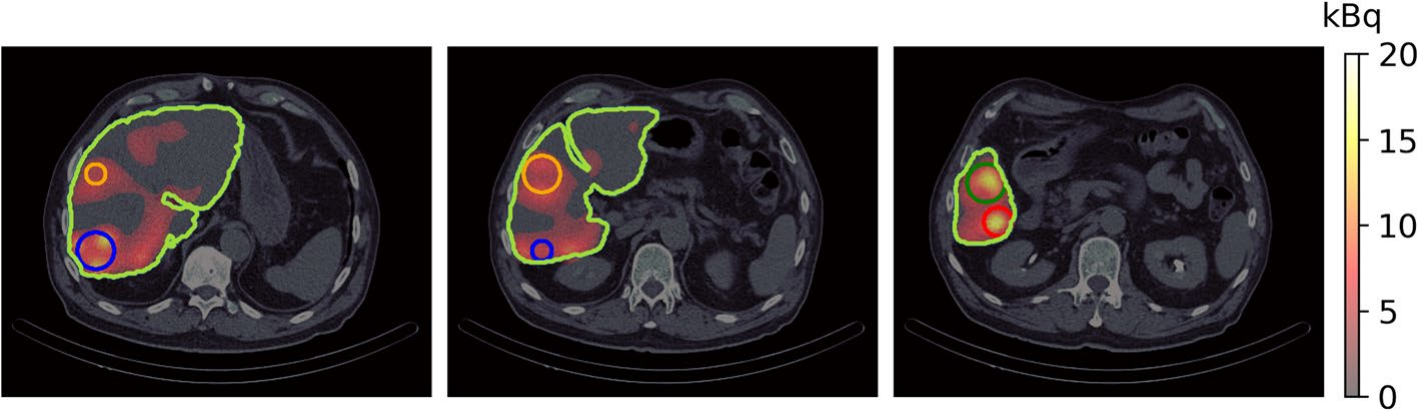
Three axial slices of fusion SPECT/CT with Liver and VOIs consider contoured

The SPECT/CT image obtained from Technetium $^{99m}$Tc albumin aggregated ($^{99m}$Tc-MAA) pretreatment simulation before $^{90}$Y hepatic radioembolization was used to calculate the absorbed dose by applying FLUKA MC and Voxel Kernel Convolution (VKC) [26]. Figure 1 shows three axial slices of the image of a patient who was administered $185 \, MBq$ of $^{99m}$Tc-MAA. The image size was $512 \times 512 \times 258$ pixels and a resolution of $0.98 \times 0.98\times 0.98\,{\text {mm}}^3$. Also, images show the segmentation of the liver and 5 VOIs. Biodistribution of $^{99m}$Tc-MAA and $^{90}$Y microsphere was considered identical.

A source routine was developed to perform the MC simulation of the absorbed dose through FLUKA introducing from external file information the position of the active voxel and the number of primary particles to be simulated proportional to the number of counts in the voxel. One hundred independent cycles of $10^8$ primary particles were simulated to achieve an acceptable level of statistical error. The CT images were transformed into a voxelized phantom to convert the HU number to material composition and mass density by calibration [56].

Monoenergetic electrons sDPK have been calculated by ML model, and then Eq. 6 was applied to obtain $^{90}$Y sDPK. A $23 \times 23 \times 23$ voxelized kernel with 1 mm$^3$ pixel size was calculated using MC volume integration of $^{90}$Y sDPK [68]. The activity map was obtained from a $^{99m}$Tc-MAA image using the equation [69]

14 $$\begin{aligned} A_{\rm voxel}\left( \ ^{90}Y\right) =\frac{A_{\rm liver}\left( \ ^{90}Y\right) \cdot C_{\rm voxel}\left( \ ^{99m}Tc\right) }{C_{\rm liver}\left( \ ^{99m}Tc\right) } \end{aligned}$$

where $C_{\rm voxel}(^{99m}Tc)$ and $C_{\rm liver}(^{99m}Tc)$ are the $^{99m}$Tc-MAA SPECT count, in the voxel and the whole liver, respectively, and $A_{\rm liver}(^{90}Y)$ is the corresponding net injected activity of $^{90}$Y, in this case 2.9 GBq.

The absorbed dose map was calculated as the convolution of the voxel cumulated activity ${\tilde{A}}(r)$ and the VDK *K*(*r*)

15 $$\begin{aligned} D(r)&= {\tilde{A}}(r) *K(r) \end{aligned}$$

16 $$\begin{aligned}&= 1.443 T_{1/2}(^{90}Y) A_{\rm voxel} *K(r) \end{aligned}$$

where $T_{1/2}$ is the $^{90}Y$ half-life ($64.2 \, h$)

The gamma index was used to compare the absorbed dose maps obtained by MC and DVK, defined as [70]

17 $$\begin{aligned} \Gamma \left( r_e,r_R\right)&=\sqrt{\frac{\Delta r^2\left( r_e,r_R\right) }{\delta r^2}+\frac{\Delta D^2\left( r_e,r_R\right) }{\delta D^2}} \end{aligned}$$

18 $$\begin{aligned} \gamma \left( r_R\right) \ {}&=\ \min \left\{ \Gamma \left( r_{e\ },r_R\right) \ \right\} \ \forall r_e \end{aligned}$$

where $\Delta r(r_e,r_R)$ is the distance between evaluated and reference points, $\Delta D(r_e,r_R)$ is the difference between doses at the evaluated and reference points, $\delta r$ is the distance difference criterion, and $\delta D$ is the dose difference criterion. The distance criterion was 3 mm, the dose criterion was $3\%$, and the dose map calculated by MC was the reference and VDK the evaluation.

## Result

### Monoenergetic sDPK

**Fig. 2:**
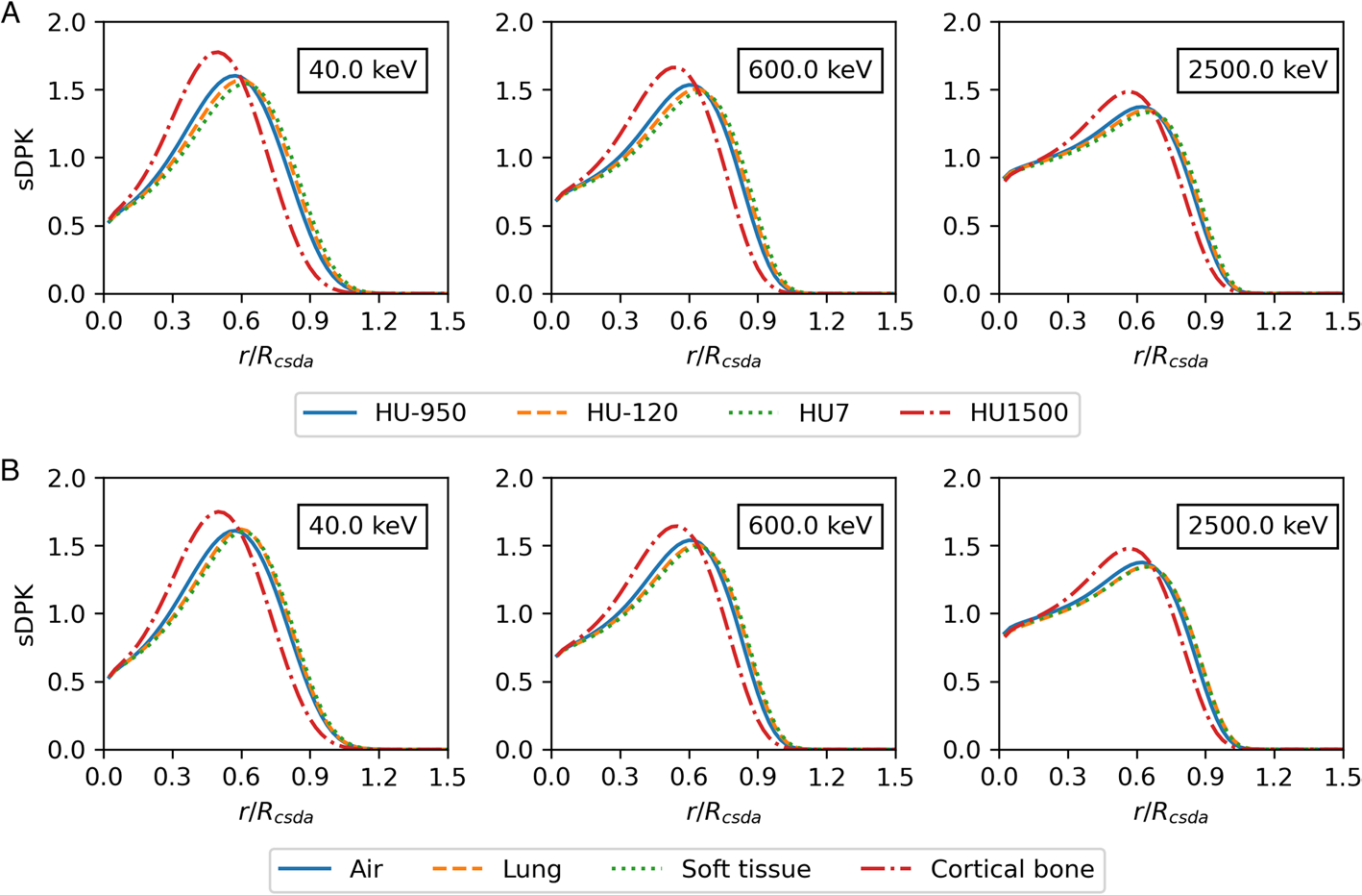
sDPK calculated by FLUKA MC for four materials from **A** the training data set and **B** the testing data set

Figure 2 shows the sDPK for a monoenergetic source of electrons obtained from calculating the energy deposited in each thickness shell at a distance by MC FLUKA and applying Eq. 7. The percentage error for each shell of sDPK was lower than $1\%$, a distance shorter than $R_{\rm CSDA}$ ($r < R_{\rm CSDA}$); when increasing the distance up to $1.2 \cdot R_{\rm CSDA}$, the percentage error increased by $4\%$. In some materials, it is found that the percentage error at distances larger than $1.2 \cdot R_{\rm CSDA}$ rises to $100\%$; therefore, longer distances have not been considered.

**Fig. 3:**
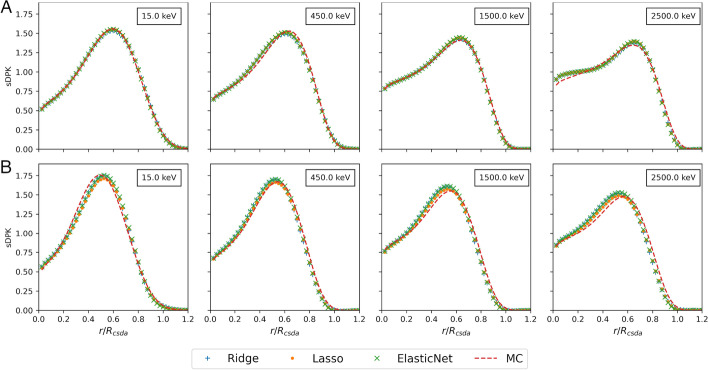
sDPK calculated by FLUKA MC and ML model for monoenergetic beta source in **A** lung, **B** compact bone. The sDPK values are plotted as a function of the scaled distance

Table 4 summarizes results for two metrics considering evaluating the performance of different base regressors models. The coefficient of determination $R^2$ reaches a value greater than 0.80 over the training set for the three base models. However, in the testing dataset, the maximum value of $R^2$ achieved 0.76 for the Lasso base regressor. The lowest value was achieved for RMSE with Ridge base regressor for training and Lasso base regressor for testing dataset; refer to Additional file 1 for more details. The sDPK estimated with the ML model for two materials of the testing set for four different monoenergetic electron sources are shown in Fig. 3.

**Table 4:** Results obtained for two metrics applied R2 and RMSE, for the three regressors considered

	Training		Testing	
	$R^2$	RMSE	$R^2$	RMSE
Ridge	**0.850(0.143)**	**0.0313(0.0144)**	0.753(0.180)	0.0385(0.0161)
Lasso	0.839(0.149)	0.0326(0.0146)	**0.766(0.184)**	**0.0367(0.0149)**
ElasticNet	0.838(0.149)	0.0329(0.0149)	0.765(0.182)	0.0369(0.0148)

Values in parentheses state for uncertainties corresponding to 1 standard deviation

### Beta-emitting radionuclide sDPK

The sDPK for $^{89}$Sr, $^{90}$Y, $^{177}$Lu, $^{186}$Re, and $^{188}$Re beta-emitting radionuclides were calculated. Emission spectra were taken from ICRP Publication 107 [71] and fitted with a smooth spline of degree 3 for each radionuclide. Then, 1000 values for pair (energy, probability) were calculated in the range of 10keV up to the maximum kinetic energy release for the radionuclide. The range of electrons for each material and energy was estimated using the analytic representation presented by Tabata et al. [65]. In the region where the ML model for calculating monoenergetic electron sDPK was valid (10 keV to 3 MeV), the analytical fit of the range showed a difference lower than $2\%$ with NIST ESTAR [72].

**Fig. 4:**
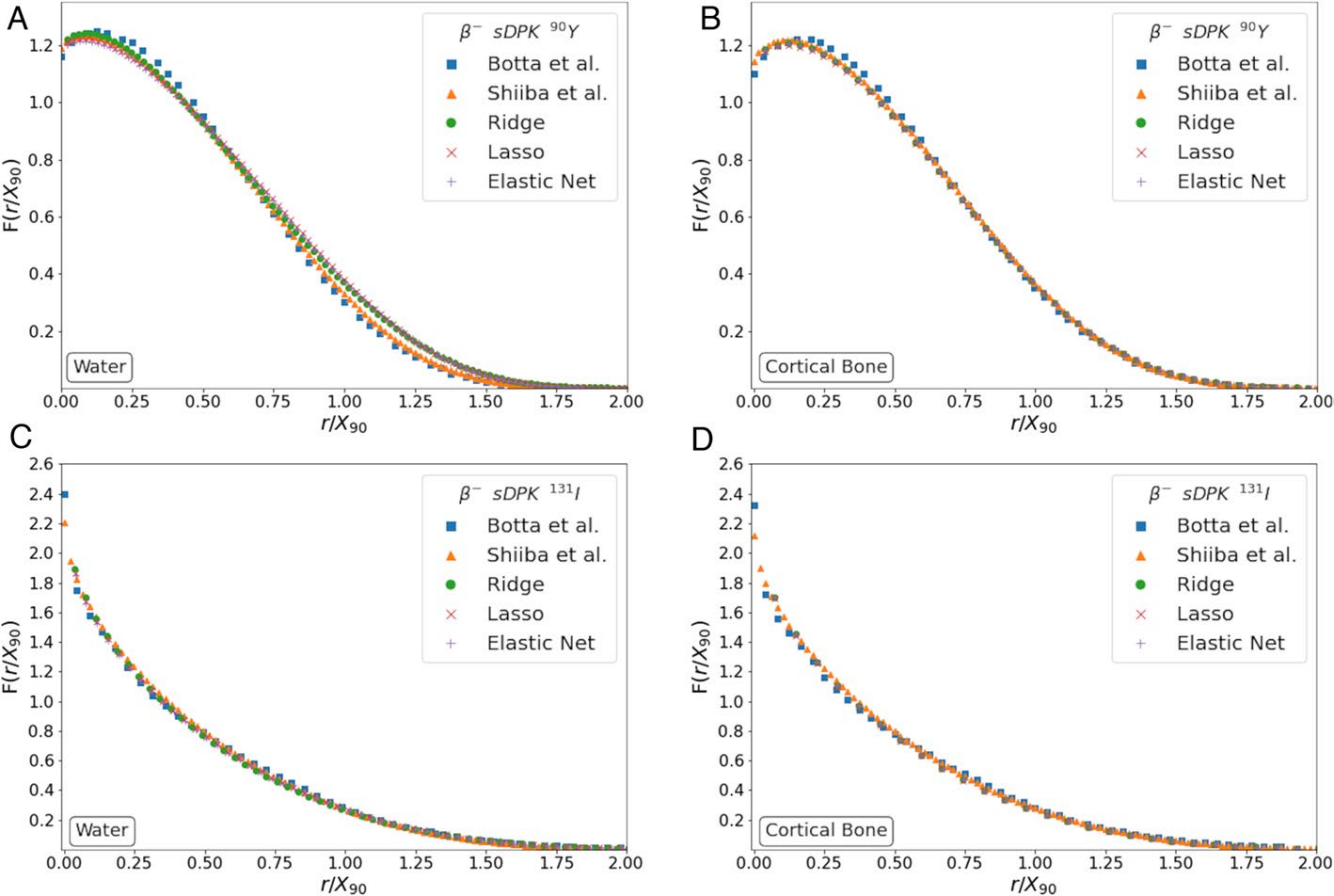
Benchmarking sDPK calculated by the ML model with the sDPK reported by Botta et al. [66] and Shiiba et al. [67]

The ML model calculates monoenergetic sDPK for each energy, the material compound according to Table 3, and the range for the material and energy considered. The beta-emitting radionuclide sDPK was determined using Eq. 6 considering a sphere of 120 shells and thickness of $r_N/100$, where $r_N$ was the range $R_{\rm CSDA}$ for the energy of maximum emission. Figure 4 shows beta-emitting radionuclide sDPK for (a) water and (b) compact bone, for $^{90}$Y and $^{131}$I, with the sDPK reported by Botta et al. [66] and Shiiba et al. [67]. It should be noted that the calculated sDPK was rescaled to the X90 scale [21]. This scale uses the distance at which $90\%$ of the emitted energy is absorbed.

### Application on dosimetry calculation

**Fig. 5:**
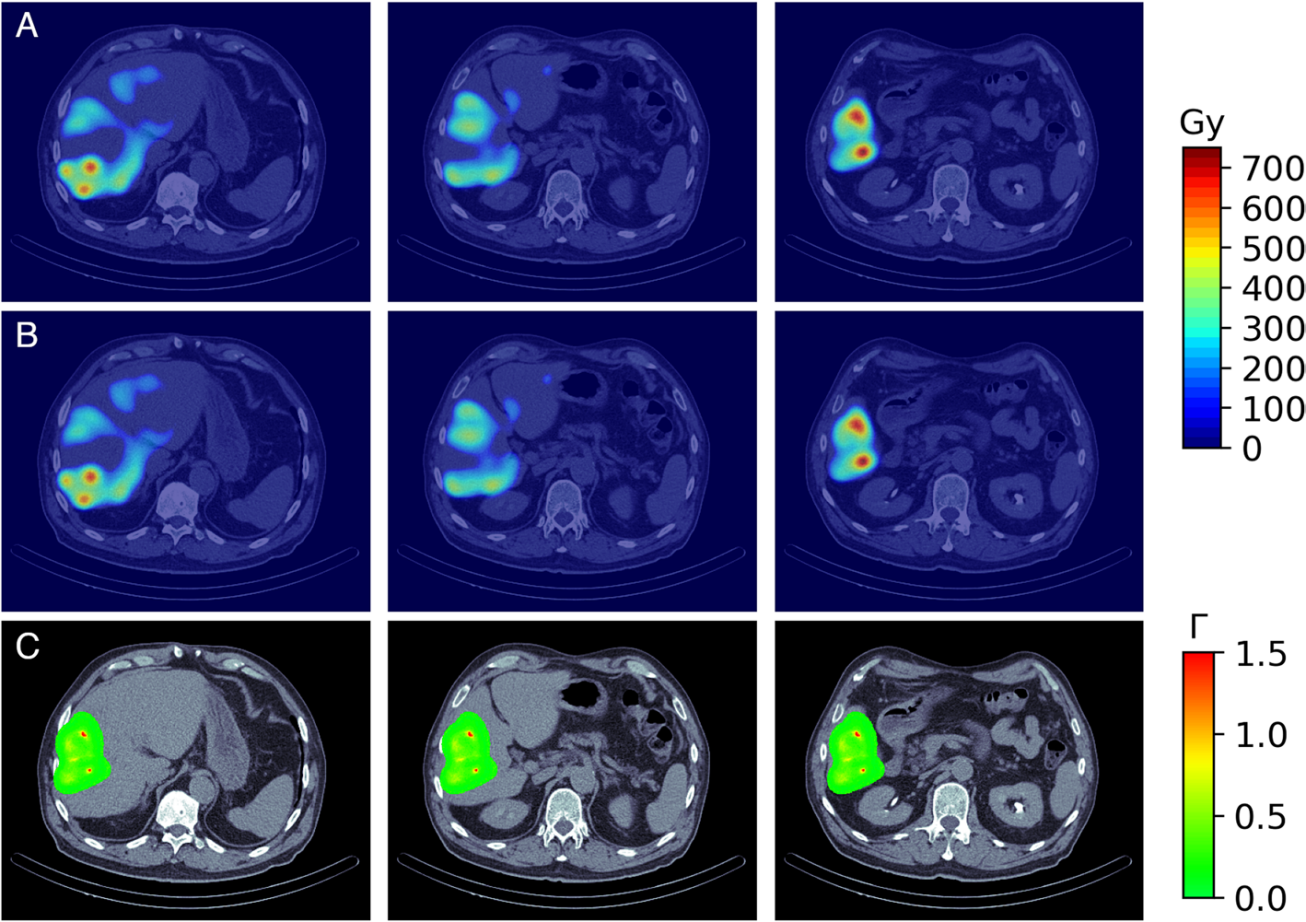
Results of dose absorbed calculated by **A** MC and **B** VDK. The figure **C** is the Index gamma for the criterion $3\,text{mm}/3\%$

Figure 5 shows the absorbed dose map calculated by (A) MC and (B) VDK for the same three axial slices shown in Fig. 1, and (C) shows the gamma index for the criterion $3\,\text {mm}/3\%$. The activity of each voxel was calculated by Eq.  14 and applied to Eq. 15. The voxelized kernel was calculated for each voxel where activity was greater than 0 using the calibration of Schneider et al. [56] to transform the Hu to the corresponding material compound. The sDPK for 90Y was calculated using Eq. 6 and the monoenergetic sDPK predicted by the RC model with Lasso as base regressor.

The mean absorbed dose calculated by MC for the liver and the five VOIs considered is greater than the mean absorbed dose calculated by VDK by approximately $7\%$. The standard deviation means of absorbed dose at voxel levels calculated by FLUKA was less than $9\%$ in the whole liver, and less than $1\%$ in 5 VOIs considered. Table 5 summarizes the results obtained for the entire liver and the five VOIs.

**Table 5:** Dose absorbed calculated by MC and DVK expressed as ${\bar{D}}\pm \sigma$ and $[D_{\rm min}, D_{\rm max}]$. Also, the gamma index calculated is reported for the Liver, and five VOIs consider

Region	Dose by MC (Gy)	Dose by DVK (Gy)	Gamma index (%)
Liver	$88.87 \pm 124.76$	$82.02 \pm 116.57$	94.96
	$[0.00, \, 728.70]$	$[0.00, \, 676.23 ]$	
VOI 1	$387.61 \pm 142.95$	$361.77 \pm 134.08$	98.19
	[25.62, 728.7]	[20.33, 676.23]	
VOI 2	$329.89\pm 148.59$	$307.61\pm 139.29$	96.01
	[2.24, 673.77]	[1.52, 627.85]	
VOI 3	$334.85\pm 83.3$	$312.69 \pm 77.41$	94.82
	[42.9, 645.47]	[40.23, 599.25]	
VOI 4	$265.95 \pm 47.29$	$248.45 \pm 44.17$	90.02
	[114.11, 398.24]	[106.17, 371.52]	
VOI 5	$205.84 \pm 41.99$	$192.52 \pm 39.2$	96.08
	[43.75, 297.07]	[40.68, 278.4]	

In more than $94\%$ of voxels, the gamma index was less than 1 for the $3\,\text {mm}/3\%$ criterion in all regions considered. Figure 5C shows gamma index maps for the same three axial slices. As can be seen, at a zone of high dose the gamma index is greater than 1.

## Discussion

The ML-based models with three different base regressors considered have been able to predict the monoenergetic electron sDPK with reasonable performance. The $R^2$ for materials in the testing dataset was more significant than 0.75 in all models studied. The performance and reliability of models decrease when the initial energy of the source increases (see Fig. 3); this is most notable in air and lung. As radionuclides commonly used in nuclear medicine present electron emission with energies lower than 2.5 MeV (e.g., $^{90}\text {Y}=2.28 \, \text {MeV}$ and $^{188}\text {Re}=2.1$  MeV), degrading performance at an energy greater than 2.5 MeV has not a significant effect when calculating the sDPK for beta-emitting radionuclide. Furthermore, the probability of emission of electrons with energies higher than 2 MeV represents a small fraction compared to the probability of emission of electrons with lower energies where the models have shown the best performance. It is worth noticing that the implemented approach based on ML algorithms’ performance to predict and evaluate sDPK may be improved to better deal with high electron energy emissions.

Table 4 shows that Lasso, as the base regressor, obtains the highest performance in the testing dataset compared to Ridge and Elastic Net. It is worth mentioning that the difference in the $R^2$ obtained between Lasso and Elastic Net is minimal. Although Ridge achieves a higher performance in the training dataset, it gives a lower result than the other two in the training dataset. This effect is due mainly to the fact that some of the features in the datasets are collinear, which generates a more significant variance in the coefficients producing that minor variations in the predictors produce significant changes in the predicted value [49]. Ridge tends to bring the regression coefficients to values close to 0. In contrast, Lasso generates a coefficients shrinkage and feature selection process intensifying the most relevant attributes and removing those that are redundant. Elastic Net produce a coefficients regularization and feature selection. So we can see that the features selection process is more robust to the features’ collinearity condition [48].

**Fig. 6:**
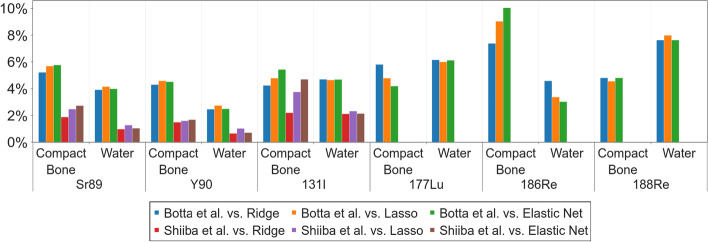
Mean absolute percentage error (MAPE) for water and compact bone, for all emitters compared to those published by Botta et al. [66] and Shiiba et al. [67]

Beta-emitting radionuclide sDPK calculated from monoenergetic sDPK estimated by ML models and using Eq. 6 showed promising agreement between values reported by Botta et al. [66] and Shiiba et al. [67] (see Fig. 5). Figure 6 shows the MAPE for compact bone and water for all radionuclides, which was less than $10\%$ compared to the sDPK reported. This discrepancy is mainly due to differences in the spectrum of emissions considered. The model for beta-emitting radionuclides sDPK does not consider the Auger and conversion electrons emitted in radionuclide decay, thus influencing at short-range level. As shown in Fig. 4, the non-negligible differences correspond to regions very close to the emission source.

Although many different compounds have been considered to calculate the sDPK database, they are limited to biological tissues. Thereby, special care is required for clinical cases involving prosthesis or implants. In this regard, further developments/extensions of the present work are planned to account for such materials. To this aim, the three ML-based models studied in this work have demonstrated the capability to quickly calculate the monoenergetic sDPK with the composition, density, energy, and range of material as input data. This allows the generation of monoenergetic sDPK easily for any material, and the beta-emitting radionuclide sDPK can be calculated by the model proposed for a wide range of radionuclides.

Finally, the application of sDPK for dosimetric calculation showed a good agreement with FLUKA MC calculation of maps of absorbed dose. Index gamma was less than 1 in more than $94 \%$ of voxels. However, voxel convolution underestimated the absorbed dose by $6\%$ approximately. The model proposed to estimate the absorbed dose by voxels kernel convolution has shown a real advantage over FLUKA MC when comparing the required calculation time, i.e., 7 min versus 40 h, respectively. Moreover, the capability of ML-based model’s to quickly calculate several monoenergetic electron sDPK was demonstrated, which were further used to estimate the beta-emitting radionuclide sDPK tailored to each material.

## Conclusion

An ML model was developed to show the capacity to accurately calculate the sDPK for monoenergetic beta sources in a wide range of energy and materials compounds. Although preliminary evaluations limited to one patient have been useful to verify the feasibility of the proposed approach as well as to suggest a promising performance, it is worth mentioning that extending the application to attain exhaustive benchmarking on a wide patient dataset remains mandatory to proceed with a definitive performance evaluation. A model for calculating the sDPK for beta-emitting radionuclide from the monoenergetic sDPK allows obtaining the VDK for calculating the absorbed dose at a patient-specific level in a short time.

Extending the proposed approach to other ML algorithms evaluating the corresponding performance may constitute a valuable future contribution.

## Supplementary Information

**Additional file 1**: Interpretation of the regressor coefficients.

## Data Availability

Anonymized $^{99m}$Tc-MAA SPECT/CT DICOM data including segmented lesions for select patients are available at the University of Michigan Library Deep Blue repository: 10.7302/v07v-z854 and 10.7302/pf4m-vn04 The datasets generated during and/or analyzed during the current study are available from the corresponding author on reasonable request.
