# Supplementary material for: A machine learning-based model for a dose point kernel calculation
Source: EJNMMI Phys. 2023 Jun 26;10:41. doi: 10.1186/s40658-023-00560-9 (PMC10293553; doi:10.1186/s40658-023-00560-9)
Supplement: Supplementary file 1 — Additional file 1: Interpretation of the regressor coefficients. [file 40658_2023_560_MOESM1_ESM.pdf]

# Interpretation of the regressor coefficients

## Multi-colinearity of features

Multicollinearity occurs when two independent variables are highly correlated. Figure 1 shows the absolute Pearson correlation coefficients for the variables in the dataset. A threshold of 0.7 was set to determine when two variables are correlated, which means they are correlated if two features have a correlation factor greater than 0.7. We can see that several characteristics meet this condition and are correlated. Finally, we can conclude that the dataset is highly collinear. Given this situation, we apply methods tolerant to this situation, such as linear regression, along with three types of penalties: Ridge (L2), Lasso (L1), and Elastic Net (L1,2).

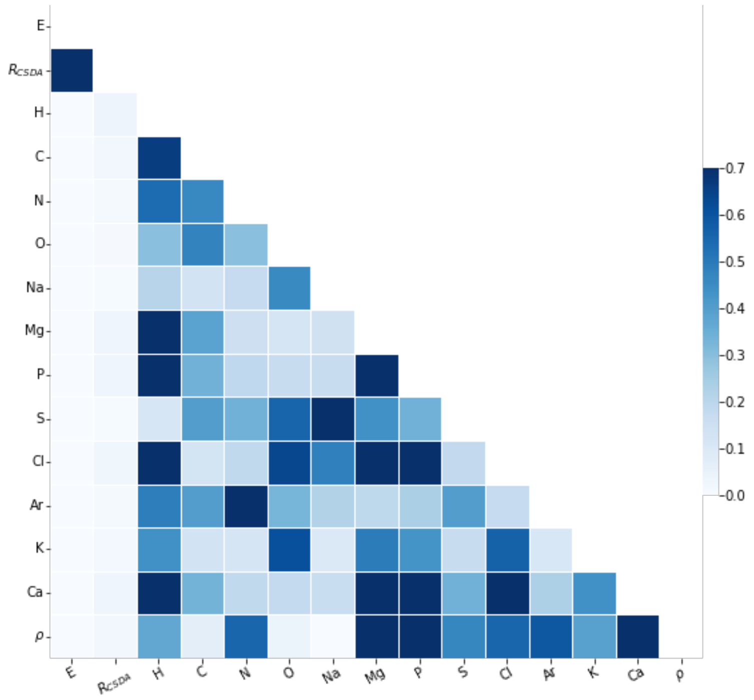


Figure 1: Absolute Pearson correlation coefficients between features of the dataset

## Analysis of the weights of estimators

The proposed model is an ensemble of chain regressors, and the linear regressor is used as the base regressor with three types of penalties applied: Ridge, Lasso, and Elastic Net. The input features are $R_{csda}$, H, C, N, O, Na,

Mg, P, S, S, Cl, Ar, K, Ca, and $\rho$ (density). We will call the estimators $D_{ri}$ where $i = 1, \ldots 48$, and the features due to the estimator $i - 1$ we will call $\tilde{D}_{ri}$ where $i = 1, 47$.

Furthermore, since all the input variables have different units of measurement for their analysis, the weight of each is multiplied by the standard deviation of the feature so that all the coefficients have the same units and can be directly compared. First, the importance of the features of the training dataset is analyzed, and then the weights of the features added in the chaining process will be examined.

Figure 2 shows the values for the coefficients of the input features corrected by the standard deviation. The energy and the $R_{csda}$ range are the most significant factors in the three cases; also, the effect of the high correlation between them can be observed, i.e., when one increases, the other decreases.

Regarding the chemical elements that compose the materials, C, N, O, and Ca have coefficients more significant than 0 in all three cases. In particular, Ridge also gives a higher weight to H, C, N, O, P, and Ca. In the case of Lasso and Elastic Net, we can see the effect of the L1 penalty, which produces a feature selection, and as can be seen in both cases, Na, Mg, S, Cl, Ar, and K are disregarded, and Elastic Net also disregards P and H. The Elastic Net model, which applies both L1 and L2 penalties, shows that in addition to selecting features, the distribution of coefficient values tends to approach 0, an effect similar to Ridge, as seen in C, N, O Ca. Then we see the impact of using the L1 and L2 norm where some factors are selected as in Lasso, and others where their values approach 0 as with RIDGE.

Figure 3 shows for each base regressor a heatmap of the values of the coefficients of the input features for the 48 estimators. The Ridge model shows that the value of the coefficients tends to decrease as the distance from the source increases, that is, for the DPK estimators for $r > 1$. On the other hand, for Lasso, it can be observed that for some features such as C, H, N, or Ca, the value tends to decrease first and then increases again between $D_r16$ and $D_{36}$. This corresponds to the range where the DPK first increases until it reaches a maximum and then decreases. In Elastic Net, the coefficients maintain their value throughout all estimators. A particular situation can be observed, as already seen in Figure 2, where the E and $R_{csda}$ coefficient values when one increases, the other decreases.

Figures 4, 5, and 6 show heatmaps of the value of the weights for the features that are consequences of the chaining process $\tilde{D}_{ri}$ for $i = 1, 47$. As seen in all three cases, the value of the coefficient of the immediately preceding feature is the most significant. In particular, unlike Ridge, Lasso, and Elastic Net give a significant value to the immediately preceding feature

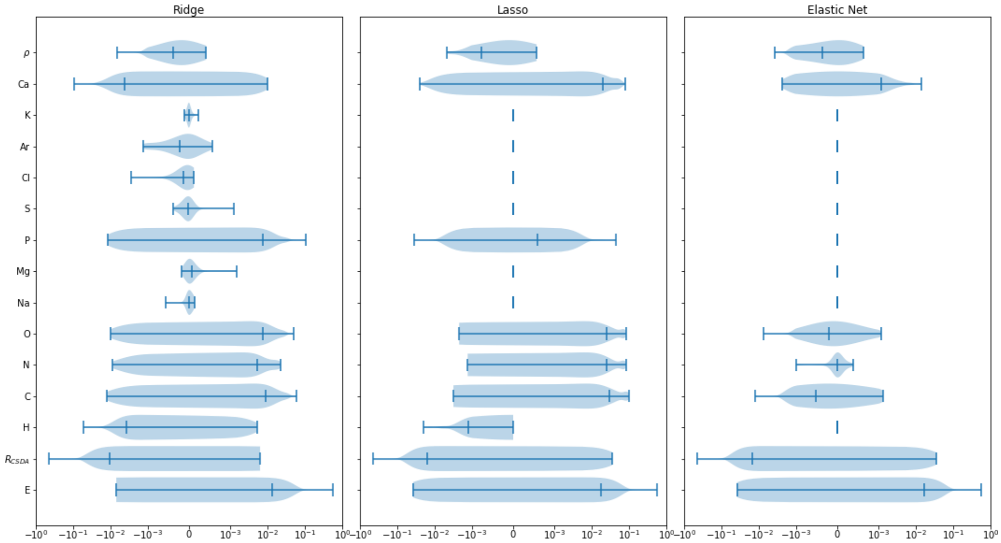


Figure 2: Weights of coefficients for the three base regressors

and give importance to previous features, moderating the priority given to the immediately preceding feature.

Figure 7 shows the weights for the coefficients for five estimators $r_0$, $r_{10}$, $r_{20}$, $r_{30}$, and $r_{40}$. As seen for the five estimators, the most significant coefficient is the one immediately before. As expected, the values of the coefficients decrease as they are farther away from the $r$ under consideration. One aspect to note is that in the case of Lasso and Elastic Net, the value of the coefficients decreases smoothly from the $r$ in question toward the $r$ closer to the source. On the other hand, in Ridge, there is an abrupt decrease with a much higher value for the immediately preceding feature than in the case of Lasso and Elastic Net.

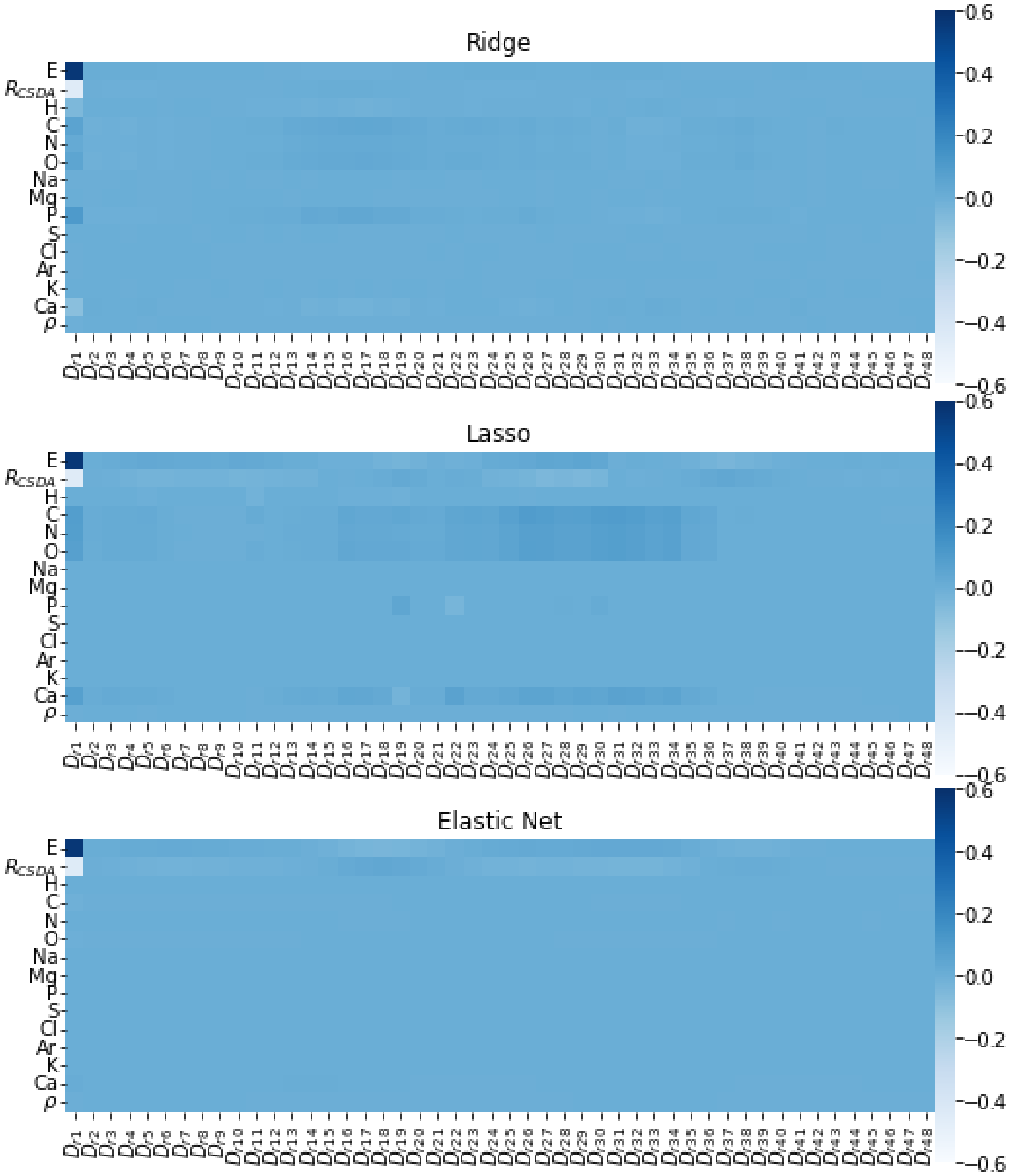


Figure 3: Heatmap of the values of the coefficients of the input features for the 48 estimators

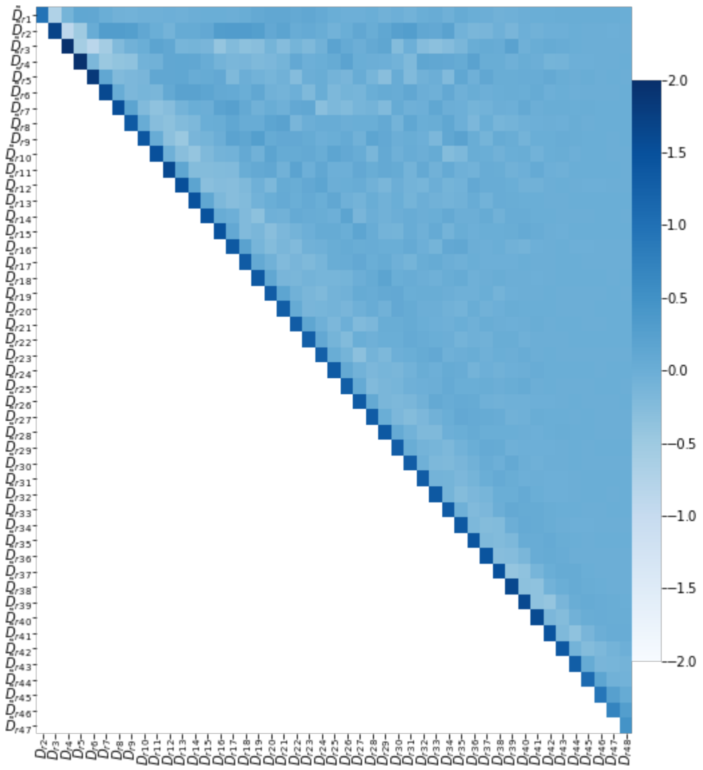


Figure 4: Heatmaps of values of the weights of coefficients of the Ridge regressor

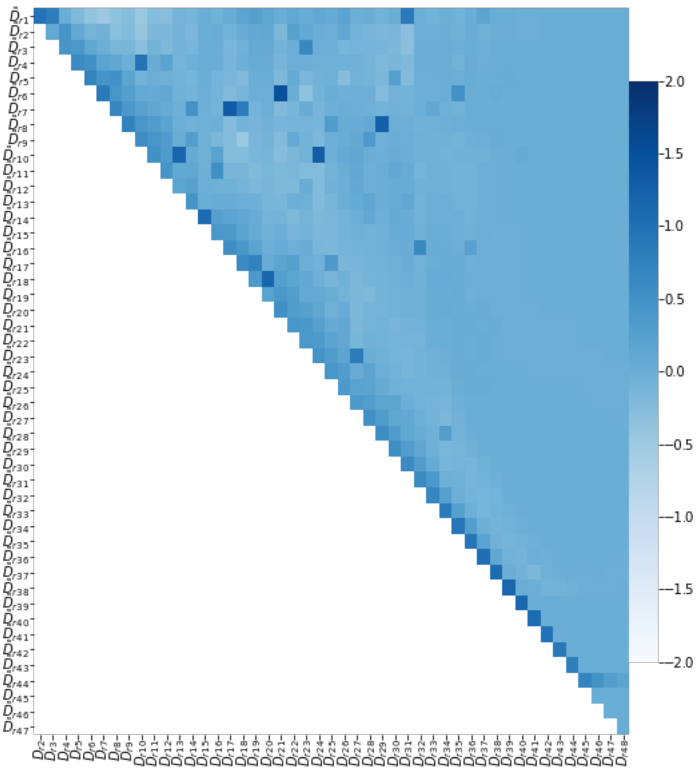


Figure 5: Heatmaps of values of the weights of coefficients of the Lasso regressor

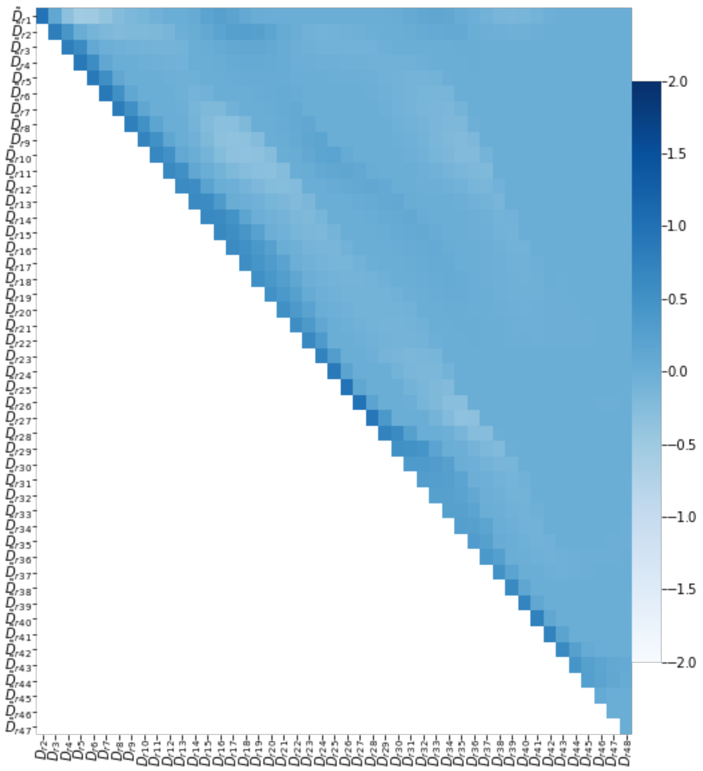


Figure 6: Heatmaps of values of the weights of coefficients of the Elastic Net regressor

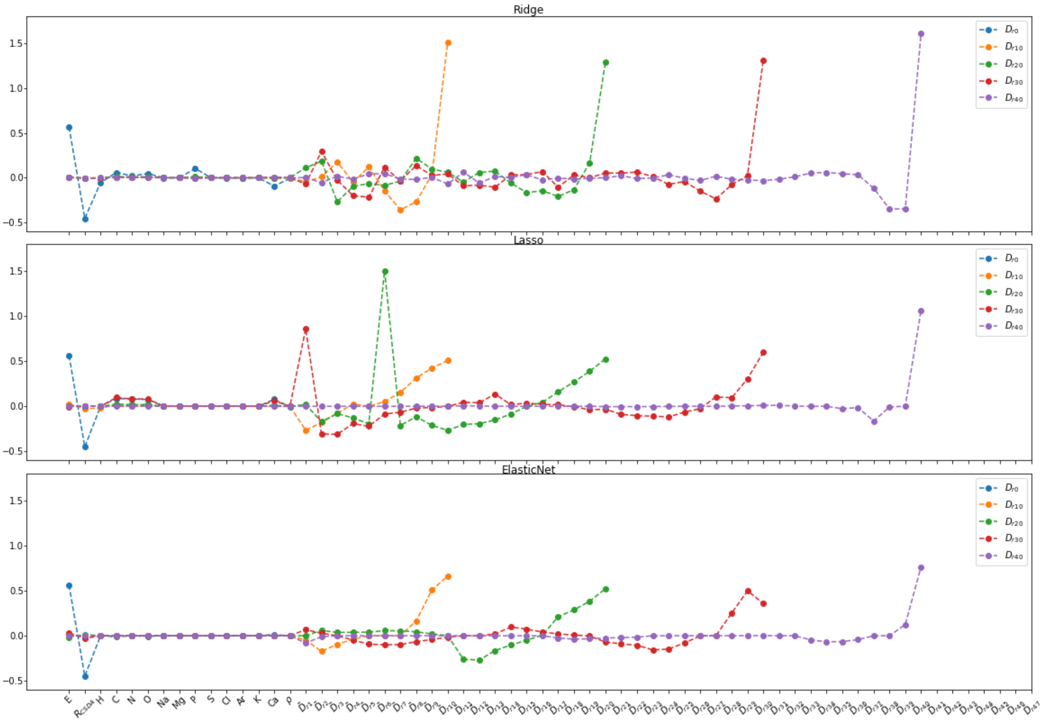


Figure 7: Coefficients para 5 estimators $r_0$, $r_{10}$, $r_{20}$, $r_{30}$, and $r_{40}$
